# Dosage of Dual-Protein Nutrition Differentially Impacts the Formation of Atherosclerosis in *ApoE^−/−^* Mice

**DOI:** 10.3390/nu14040855

**Published:** 2022-02-18

**Authors:** Yingchun Huang, Kun Zhang, Li Zhang, Juhui Qiu, Lin Fu, Tieying Yin, Jing Wang, Rui Qin, Jingjie Zhang, Xianwen Dong, Guixue Wang

**Affiliations:** 1 Key Laboratory for Biorheological Science and Technology of Ministry of Education, State and Local Joint Engineering Laboratory for Vascular Implants, College of Bioengineering, Chongqing University, Chongqing 400044, China; hyc97cy@163.com (Y.H.); kunzh01@163.com (K.Z.); jhqiu@cqu.edu.cn (J.Q.); tieying_yin@cqu.edu.cn (T.Y.); 2 Chongqing Academy of Animal Sciences, Chongqing 402493, China; zhangli03094@163.com (L.Z.); lyfl1990@163.com (L.F.); 3 Institute of Food and Nutrition Development, Ministry of Agriculture and Rural Affairs, Beijing 100086, China; jingjiezhang@163.com; 4 College of Life Sciences, South-Central University for Nationalities, Wuhan 430079, China; qinrui@scuec.edu.cn

**Keywords:** atherosclerosis, dual proteins, dosage, lipid metabolism, gut microbiota

## Abstract

Atherosclerosis (AS) is recognized as the original cause of most cardiovascular and cerebrovascular diseases. The dual-protein (DP) nutrition that consists of soy protein and whey protein is reported to be associated with a reduction in AS; however, the relationship between DP and AS remains ambiguous. Therefore, this study aimed to verify the effect of DP on AS and explore the optimal DP intake to improve AS. *ApoE^−/−^* mice were administrated with low- (LDP), middle- (MDP), and high-dose (HDP) DP. The MDP group exhibited significant improvements in AS. In terms of lipid metabolism, the levels of plasma total triglyceride and LDL-C and the mRNA expression levels of Cyp7a1 and PCSK9 were markedly tuned in the MDP group. In addition, the MDP treatment group had a substantially lower inflammatory response and better intestinal barrier function than LDP and HDP groups. The species richness demonstrated by the Chao1 index was distinctly increased in the MDP group, and the relative abundance of intestinal-permeability-protective microbes *Blautia* and *Akkermansia* was significantly elevated. In summary, an adequate intake of DP was able to counteract atherosclerosis development in *ApoE^−/−^* mice, and this study provides a scientific theoretical basis for the application of DP in the food and pharmaceutical fields.

## 1. Introduction

Atherosclerosis (AS) is a common cardiovascular disease (CVD) that involves the arterial wall and is characterized by the progressive accumulation of lipids and inflammatory cells in the intima of large arteries, which is still a major reason for mortality all over the world [1]. The pathogenesis of AS is not fully known, and atherogenesis involves a variety of risk factors including hyperlipidemia, hypertension, diabetes, smoking, and drinking [2]. Previous studies have reported that inflammatory response and the disorder of lipid metabolism are closely related to atherosclerosis [3], while poor dietary choices such as Western diet can also contribute to an increased risk of CVD, specifically AS [4]. Currently, treatment options for AS can be divided into interventional and noninterventional methods [5]. In the last few decades, epidemiological, clinical, and experimental studies have demonstrated that dietary intervention as a noninterventional therapy plays a central role in the prevention of AS [6]. The lipid-lowering diet has been revealed to be effective in regressing atherosclerotic plaque [7]. Clinical studies have shown that the Mediterranean diet is capable of reducing blood pressure, inflammation, and atherosclerosis [8]. Therefore, it is critical to identify the impact of dietary interventions on atherosclerosis.

Sufficient dietary protein is essential to maintain human health. The dual-protein (DP) powder in this study mainly consists of soy protein isolate and whey protein, which proved to be useful for human health, including CVD [9]. In a randomized parallel-group study of 44 type 2 diabetes mellitus patients (T2D), supplementation with high animal protein or high plant protein diets significantly decreased the total and LDL cholesterol levels and systolic blood pressure, as well as the cardiovascular risk, of patients with T2D [10]. Aside from the improvement of CVD, in a rat exercise study, intake of soy–whey blended protein was found to improve the sports performance of rats while enhancing the activities of lactate dehydrogenase and superoxide dismutase in serum and decreasing the levels of malondialdehyde in serum [11]. Soy or whey protein supplements also improved lipid levels in hypercholesterolemic men [9].

High protein plays a key role in preventing obesity, high blood pressure, and T2D [12,13]; it was even shown that a high-protein diet significantly improved the body weight and lipid profiles of diabetic patients compared to a low-protein diet [14]. However, some studies have reported that the high-protein diet is associated with an increased risk of CVD [15,16]. There are also some studies on the benefits of low-protein diets. Kalantar-Zadeh et al. reported that a low-protein diet may regulate the production of uremic toxins and slow down the progression of chronic kidney disease (CKD) through beneficial alterations in gut microbes while reducing cardiovascular risk [17]. In a study considering 38 patients with various kidney diseases, after their dietary protein intake was decreased from ≥1.0 g/kg/day to 0.6–0.7 g/kg/day, insulin resistance of patients was improved remarkably, which was the major risk factor of atherosclerosis [18]. In summary, it is speculated that the dosage of protein intake is the key point causing the controversial impacts. Hence, in this study, we set different doses of DP to explore the best dose to improve AS (basis of CVD) in *ApoE^−/−^* mice, so as to provide a new dietary intervention strategy for improving AS.

## 2. Materials and Methods

### 2.1. Food Supplement and Diet

The dual-protein (DP) food nutrition powder used in this experiment was a finished product, which was developed by the Food and Nutrition Development Research Institute of the Ministry of Agriculture and produced by Competitor Sports Science Technology Joint Stock Co., Ltd (Beijing, China). The product was mainly made of soy protein isolate and whey protein as the main food nutrition base material, supplemented with dietary fiber and functional nutrients. The percentages of soy protein isolate and whey protein are 48% and 32%, respectively. The product is dedicated to dual-protein engineering with Chinese characteristics and contains 31.5% carbohydrates, 52.5% protein, and 6.5% fat. Mice raised on a normal-chow diet were purchased from Jiangsu Xietong Pharmaceutical Bio-engineering Co., Ltd., (Jiangyin, China). The normal-chow diet contained 67.4% carbohydrates, 20.6% protein, and 12.0% fat, while the high-fat diet purchased from Changzhou SYSE Bio-Tec Co., Ltd., (Changzhou, China), contained 40% carbohydrates, 20% protein, and 40% fat. All animal diets in this experiment conform to the animal food standard.

### 2.2. Animal Raising and Modeling

The ApoE-knockout mouse model for atherosclerosis was generated on a C57BL/6JGpt genetic background using CRISPR-Cas9. The *ApoE^−/−^* mouse is a well-established model for the study of the formation and progression of AS [19]. Forty-five male *ApoE^−/−^* mice (seven weeks old, 20 ± 2 g) were purchased from the company called GemPharmatech Co., Ltd., (Jiangsu, China). All the mice had to acclimate to the new environment for a week before the experiment. The mice were kept in a strictly controlled condition with a temperature of 22 ± 2 °C and the humidity of 50–60%, alternating day and night for 12 h, and allowed to eat and drink freely. Animals were raised and treated following national ethical guidelines.

After a week of acclimatization, the mice were randomly divided into five groups (*n* = 9 per group): normal-chow-diet group (NCD + phosphate-buffered saline (PBS), oral gavage daily of 0.2 mL PBS for 12 weeks), high-fat-diet group (HFD + PBS, oral gavage daily of 0.2 mL PBS for 12 weeks), the low-dose DP gavage group (HFD + LDP, oral gavage daily of 0.33 g/kg/day DP for 12 weeks), the middle-dose DP gavage group (HFD + MDP, oral gavage daily of 0.67 g/kg/day DP for 12 weeks), and the high-dose DP gavage group (HFD + HDP, oral gavage daily of 1.33 g/kg/day DP for 12 weeks). The dose design was based on the manufacturer’s recommended daily intake of 10–20 g/60 kg BW. Meanwhile, we combined the method of equal ratio calculation to set three concentrations.

The body weights of mice were measured at 9:00 a.m. every Monday until the end of the gavage. The daily food intake of mice was measured for five consecutive days before the end of animal modeling.

### 2.3. Tissue Sampling

At the end of modeling, all the mice were sacrificed under anesthesia and the blood was collected in an anticoagulant tube. Plasma was prepared under centrifugal conditions (4 °C, 3000 rpm for 10 min) and stored at −80 °C. Then, the whole aorta, heart, liver, ileum, white fat, and cecum contents of the mice were collected. Part of the tissue was fixed with 4% paraformaldehyde fix solution and stored at 4 °C for staining analysis, while the other part was stored at −80 °C for further experiments. All animal experiments were approved by the Chongqing University Ethics Committee.

### 2.4. Biochemical and Immunological Assays of Plasma

The levels of plasma total triglyceride (TG), total cholesterol (TC), high-density lipoprotein cholesterol (HDL-C), and low-density lipoprotein cholesterol (LDL-C) were detected by the Chemray 240 automatic biochemical analyzer (Servicebio Technology Co., Ltd., Wuhan, China). The inflammatory factors IL-1β and TNF-α in plasma were detected by ELISA kit (Catalog Numbers 88-7324 and 88-7013, respectively, Thermo Scientific Inc., MA, USA). All the experimental procedures were carried out according to the instructions.

### 2.5. Atherosclerotic Lesion Analysis

Oil Red O staining was used to estimate atherosclerotic lesions, including the aorta and the aortic root. The aortic region is from the ascending aorta to the descending aorta. The aortic root was sliced into 10 µm thick serial sections from the aortic sinus to the aortic arch. The lesion area was the red-stained area, which was analyzed by the software of ImageJ (ImageJ 1.53e, Wayne Rasband, National Institutes of Health, Bethesda, MD, USA). To reduce the error caused by the size of the tissue, the result is expressed as the ratio of the stained area to the total tissue area.

### 2.6. Morphologic and Histology Analysis

To determine the lipid deposition of the liver, Oil Red O staining was used to analyze liver lesions. The liver fixed with 4% paraformaldehyde was dehydrated, embedded by OCT, sliced into serial liver sections, and finally stained with Oil Red O. The paraffin sections of the adipose tissue were stained with hematoxylin and eosin for observing the size and morphology of adipocytes after the removal of embedding agent and rehydration. For verifying the effect of DP on the intestinal barrier, the Alcian blue staining of the ileum tissue was detected and identified by the mucus layer and goblet cells. The paraffin sections of the ileum were stained with Alcian blue dye solution A and dye solution B; then, sections were dehydrated and sealed with neutral resin, and images were taken under the microscope. The lesion area was analyzed by the software of ImageJ. The diameter of the adipocyte was measured by ImageJ. The number of stained goblet cells in ileum tissue sections was quantified by the counting tool in the software of ImageJ.

### 2.7. Immunofluorescent and Immunohistochemical Assay

For the immunofluorescent assay, the prepared frozen sections of aortic root were washed with PBS (pH 7.4) three times to remove OCT (5 min each) and then covered with 5% BSA for 30 min to block nonspecific binding, followed by incubation with anti-intercellular adhesion molecule-1 (ICAM-1; 1:200; Santa Cruz Biotechnology, Inc., Dallas, TX, USA) or anti-CD68 (ZO-1; 1:400; Abcam Inc., Cambridge, UK) antibodies. The ileal sections were incubated with anti-zonula occludens-1 (ZO-1; 1:100; Abcam Inc., Cambridge, UK) or occludin (occluding; 1:100; Abcam Inc., Cambridge, UK) antibodies. The incubation conditions for the primary antibody were overnight at 4 °C. Besides, sections were incubated with secondary antibodies conjugated with Alex Fluor 555 and counterstained with DAPI (Beyotime Co., Ltd., Shanghai, China). Images were collected by laser scanning confocal microscope for immunohistochemistry assay.

Frozen sections were washed with PBS (pH 7.4) to remove OCT as described previously. Then, the sections were subjected to antigen retrieval by antigen retrieval buffer. After the endogenous peroxidase activity had been inhibited by 3% hydrogen peroxide for 25 min, sections were incubated overnight at 4 °C with vascular cell adhesion protein 1 (VCAM-1; 1:200; Santa Cruz Biotechnology Inc., Dallas, TX, USA). The sections were covered with secondary antibody (HRP-labeled) and incubated at room temperature for 60 min, and then diaminobenzidine was used to represent the target area and the nucleus was counterstained with hematoxylin. Images were obtained by optical microscope. The positive staining area was counted by ImageJ and the percentage of positive area in the total area was calculated.

### 2.8. RT-qPCR Analysis of Particular mRNA Levels in Liver

Total RNA was isolated from liver or ileum using Trizol reagent (Thermo Scientific Inc., Waltham, MA, USA), tissues were homogenized on ice, organic and inorganic phases were separated with chloroform, supernatants obtained by centrifugation at 12,000× *g* for 15 min at 4 °C were precipitated with isopropyl alcohol, and precipitates were washed with 75% ethanol and redissolved with DEPC water. The cDNA was synthesized using the PrimeScript RNA Transcription Reagent Kit with gDNA Eraser according to the manufacturer’s instructions (TaKaRa Inc., Tokyo, Japan) [20]. Real-time qPCR was performed by using the SYBR Green RT-qPCR procedure. The total volume of the RT-qPCR reaction was 20 µL, including 1 µL cDNA, 1 µL forward primer, 1 µL reverse primer, 7 µL water, and 10 µL TB Green Premix Ex Taq II (Tli RNaseH Plus) (TaKaRa Inc., Tokyo, Japan). All samples were repeated in triplicate. Glyceraldehyde-3-phosphate dehydrogenase (GAPDH) was used as an internal reference, which normalizes the expression of the target gene using the comparative CT method. All the primers were designed by using Primer-blast (the primer designing tool of NCBI). All primer sequences are listed in Table 1.

### 2.9. Glucose Tolerance Test

After an overnight (12 h) fasting period, 0.2 mL glucose (2 g/kg body weight) was intraperitoneally injected. The blood samples were obtained by tail-tip bleeding, and the blood glucose levels were measured at 0, 30, 60, 90, 120, and 180 min time points using a blood glucose meter (Sinocare Inc., Changsha, China).

### 2.10. 16S rRNA Sequencing of Cecal Contents

Eight samples were randomly selected from each group for DNA sequencing analysis to obtain the gut microbiota profiles. Total genome DNA from samples was extracted using CTAB/SDS method, and the DNA was diluted to 1 ng/µL with sterile water based on the concentration of DNA [21]. Then, 16S/18S rRNA genes were amplified using the specific primer with the barcode (primer: 16S V4-V5: 515F-907R, 18S V9: 1380F-1510R, ITS1: ITS1F-ITS2R), and the PCR products were further quantified and identified. Then, the mixture of PCR products was purified with Gene JET Gel Extraction Kit (Thermo Scientific Inc., Waltham, MA, USA). Sequencing libraries were generated using NEB Next Ultra DNA Library Prep Kit for Illumina (NEB Inc., Beijing, China) following the manufacturer’s recommendations, and the library quality was assessed on the Qubit 2.0 Fluorometer (Thermo Scientific Inc., Waltham, MA, USA) and Agilent Bioanalyzer 2100 system [22]. The library was sequenced on an Illumina MiSeq platform and 250/300 bp paired-end reads were generated. At last, the sequencing results were analyzed by bioinformatics. For instance, alpha diversity and principal coordinates analysis (PCoA) calculations were performed using the R package “qiime” (Vienna, Austria). Operational taxonomic unit (OTU), nonmetric distance scaling (NMDS), and taxonomic analysis calculations were performed using the R package “vegan”.

### 2.11. Statistical Analysis

GraphPad Prism 7.0 (GraphPad Software) was employed for statistical analyses. All result values were represented as mean ± SD. The KS normality test was used to estimate the normality of data distribution. One-way analysis of variance (ANOVA) was applied to analyze the statistical differences between the groups, and then the Tukey post hoc test was used for multiple comparisons. For non-normally distributed values, Kruskal–Wallis test was performed and followed by Dunn’s multiple comparison test. All *p* values were two-tailed, and *p* < 0.05 was considered statistically significant.

## 3. Results

### 3.1. Oral Treatment with MDP Alleviated High-Fat-Diet-Induced Atherosclerotic Lesion of ApoE^−/−^ Mice

**Figure 1:**
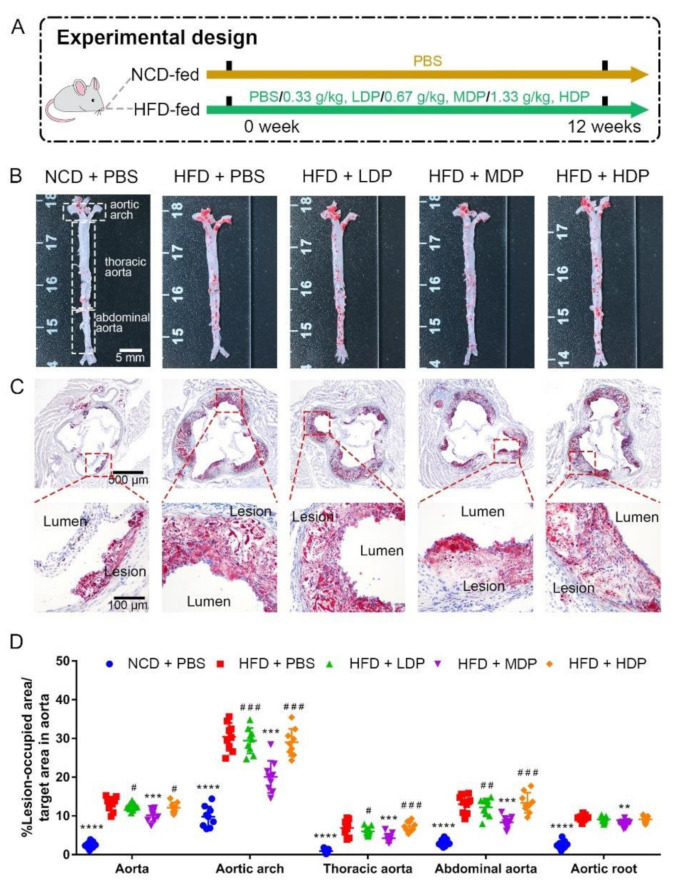
Middle-dose dual-protein (MDP) administration alleviated the progression of atherosclerosis in *ApoE^−/−^* mice. (**A**) Experimental design. *ApoE^−/−^* mice were divided into 5 groups and received different gavage treatments. (**B**) Representative images of plaque lesions in the whole area of the aorta stained with Oil Red O. (**C**) Representative of Oil Red O staining of the aorta root sections. (**D**) Quantitative analysis of lesion area in aorta, aortic arch, thoracic aorta, abdominal aorta, and aorta root sections. Statistical significance is indicated by asterisk (*) when comparing to HFD + PBS group and by hash mark (#) when comparing the other high-fat-diet-fed groups to HFD + MDP group. Data are presented as mean ± SD. *n* = 9, ** *p* < 0.01, *** *p* < 0.001, **** *p* < 0.0001, # *p* < 0.05, ## *p* < 0.01, ### *p* < 0.001. Phosphate-buffered saline (PBS), normal-chow-diet (NCD), high-fat-diet (HFD), low-dose dual-protein (LDP), high-dose dual-protein (HDP).

*ApoE^−/−^* mice were treated with DP through oral gavage daily for 12 weeks (Figure 1A). The effect of DP on plaque development was assessed by measuring red oil-stained lipid areas within the aortas and the aortic roots among the NCD + PBS, HFD + PBS, HFD + LDP, HFD + MDP, and HFD + HDP groups (Figure 1B,C). HFD + PBS group resulted in a 5.4-fold and 3.7-fold increase in aortic and aortic root lesion area in *ApoE^−/−^* mice compared with the NCD + PBS group (Figure 1D). In comparison with the HFD + PBS group, oral treatment with the MDP substantially reduced the lesion area of the aorta and the aortic root by 23% and 16%, respectively, whereas the lesion areas in the HFD + LDP and HFD + HDP groups were not significantly (*p* > 0.05) changed (Figure 1D), indicating the alleviative effect of the MDP on atherosclerotic lesions.

**Figure 2:**
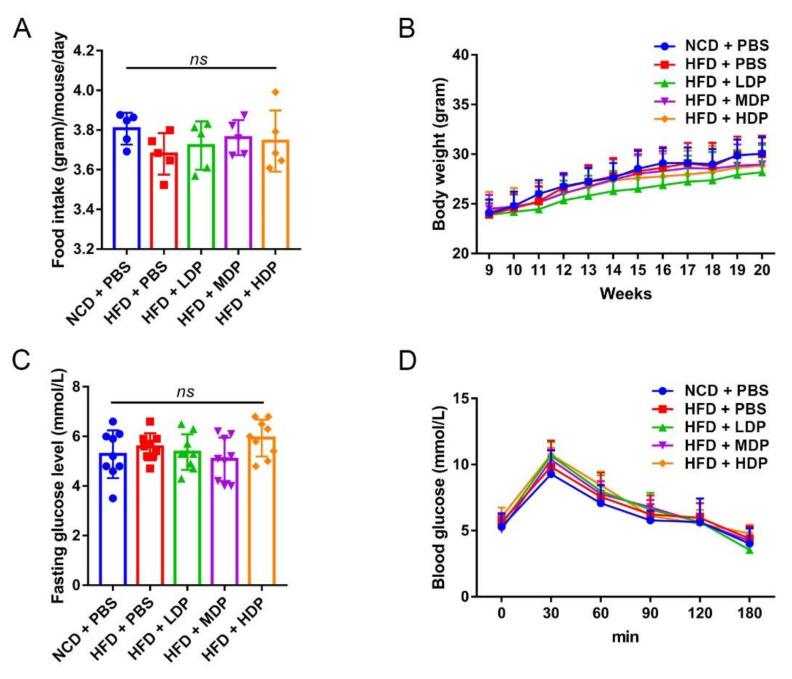
The effects of dual-protein (DP) nutrition on physiological conditions of high-fat-diet-fed *ApoE^−/−^* mice. *ApoE^−/−^* mice were grouped and treated as in Figure 1. (**A**) Daily food intake was recorded for five consecutive days and the average daily food intake was calculated. (**B**) Body weight was recorded once a week throughout the experimental period. (**C**) Fasting blood glucose level was measured after fasting for 12 h. (**D**) Glucose tolerance test was performed after 11 weeks of DP administration. Data are presented as mean ± SD. *n* = 9, *ns* indicates no significance. Phosphate-buffered saline (PBS), normal-chow-diet (NCD), high-fat-diet (HFD), low-dose dual-protein (LDP), middle-dose dual-protein (MDP), high-dose dual-protein (HDP).

Basic physiological conditions of *ApoE^−/−^* mice were recorded throughout the experimental period. A significant difference in food intake was not observed among the five groups (Figure 2A). Although the body weight of each group of mice increased from the 9th week to the 20th week, there was no significant difference in the body weight among the five groups of mice at different time points (Figure 2B). In addition, DP intervention did not greatly affect glucose tolerance or fasting blood glucose level in *ApoE^−/−^* mice (Figure 2C,D), suggesting that the beneficial effect of MDP on atherosclerosis was not attributed to altered glucose metabolism.

### 3.2. Oral Gavage with MDP Improved Lipid Metabolism in ApoE^−/−^ Mice

**Figure 3:**
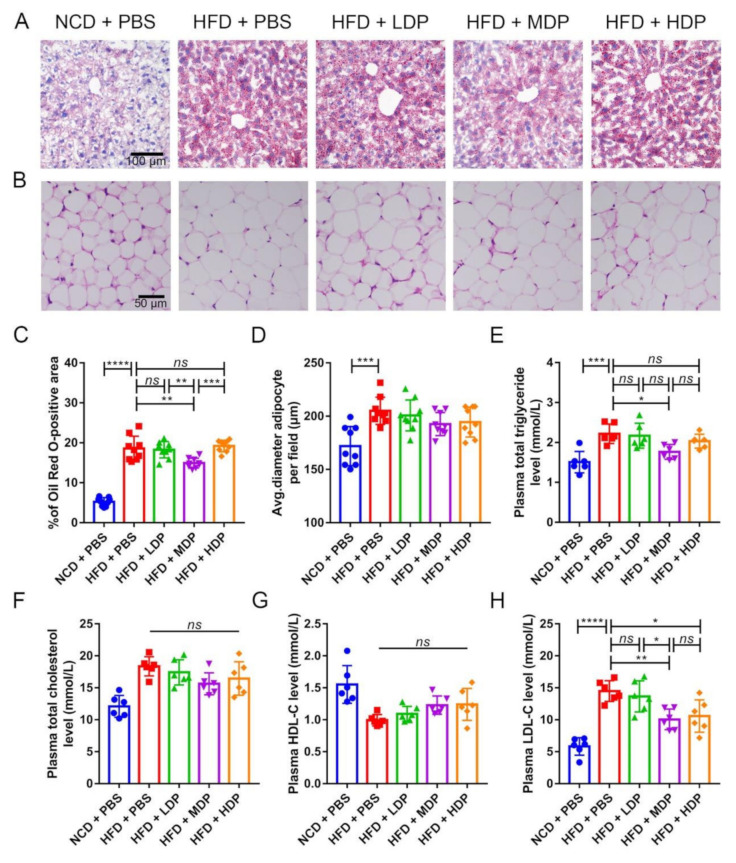
Oral treatment with middle-dose dual-protein (MDP) nutrition alleviates high-fat-diet-induced abnormal lipid metabolism in *ApoE^−/−^* mice. *ApoE^−/−^* mice were grouped and treated as in Figure 1. (**A**) Representative images of Oil Red O staining of liver sections. (**B**) Representative images of HE staining of adipocytes. (**C**) Percentage of Oil Red O positive area was calculated from (**A**) by Image J software. (**D**) Quantitative analysis of adipocyte size from (**B**) by Image J software. (**E**–**H**) Plasma TG, TC, HDL-C, and LDL-C were measured after fasting for 12 h. Data are presented as mean ± SD. *n* = 6 to 9, * *p* < 0.05, ** *p* < 0.01, *** *p* < 0.001, **** *p* < 0.0001, *ns* indicates no significance. Phosphate-buffered saline (PBS), normal-chow-diet (NCD), high-fat-diet (HFD), low-dose dual-protein (LDP), high-dose dual-protein (HDP).

The effect of DP on lipid metabolism in *ApoE^−/−^* mice was investigated since dyslipidemia is an important risk factor for atherosclerosis [3]. Lipid deposition in the liver of *ApoE^−/−^* mice was observed by Oil Red O staining (Figure 3A). It was found that the *ApoE^−/−^* mice fed with a high-fat diet had varying degrees of lipid deposition. Compared with the HFD + PBS group, the proportion of lipid droplets (Oil Red O staining) in the HFD + MDP group was significantly reduced by 19.75%, while the HFD + LDP group and HFD + HDP group were not improved (Figure 3A,C). White adipocytes were observed by HE staining (Figure 3B). The size of adipocytes in *ApoE^−/−^* mice increased by 16.15% in the HFD + PBS group compared to the NCD + PBS group, while DP intervention did not affect the adipocyte size (Figure 3D). The blood lipid level of each group was determined since it has been reported that a high-fat diet can lead to hyperlipidemia in *ApoE^−/−^* mice compared with a normal-chow diet [19]. Treatment with MDP resulted in a 20.47% reduction in TG level, but the other two doses of DP did not significantly change the TG level (Figure 3E). Plasma TC, HDL-C, and LDL-C levels were also detected, and significant differences were not found in TC and HDL-C (Figure 3F,G); however, the level of LDL-C in the HFD + MDP and HFD + HDP, especially in HFD + MDP, decreased significantly (Figure 3H). In sum, MDP can significantly reduce plasma TG and LDL-C levels, indicating that MDP was better than LDP and HDP in regulating lipid metabolism.

**Figure 4:**
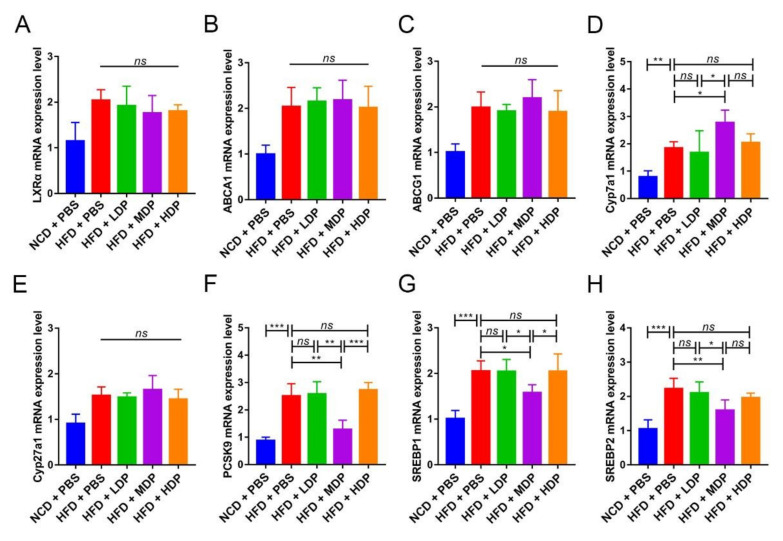
Middle-dose dual-protein (MDP) nutrition regulated the relative expression of lipid metabolism-related genes of *ApoE^−/−^* mice. *ApoE^−/−^* mice were grouped and treated as in Figure 1. (**A**–**H**) Representative gene expression levels for LXRα, ABCA1, ABCG1, Cyp7a1, Cyp27a1, PCSK9, SREBP1, and SREBP2 in the liver were quantified. Data are presented as mean ± SD. *n* = 6, * *p* < 0.05, ** *p* < 0.01, *** *p* < 0.001, *ns* indicates no significance. Phosphate-buffered saline (PBS), normal-chow-diet (NCD), high-fat-diet (HFD), low-dose dual-protein (LDP), high-dose dual-protein (HDP).

The relative expression of lipid metabolism-related genes was measured in the liver of *ApoE^−/−^* mice. LXRα regulates cholesterol reversal by regulating downstream genes such as ABCA1 and ABCG1, which are mainly expressed in the liver [23]. The mRNA expression levels of LXRα, ABCA1, and ABCG1 were quantified, and significant differences were not observed among high-fat-diet-fed groups (Figure 4A–C). However, Cyp7a1, the downstream gene of LXRα and a regulator of the production of bile acids, was remarkably upregulated in the HFD + MDP group compared to the HFD + PBS group (*p* < 0.05) (Figure 4D). Cyp27a1, which is mostly involved in the alternative pathway, was not affected by DP treatment (Figure 4E). PCSK9 is involved in LDL-C metabolism as an independent factor of high plasma LDL-C level [24]; MDP significantly decreased PCSK expression compared to other added high-fat-diet-fed groups (*p* < 0.05) (Figure 4F). Compared with the HFD + PBS group, the expression levels of SREBP1 and SREBP2 were also significantly decreased in the HFD + MDP group. Hence, MDP may accelerate cholesterol metabolism by upregulating Cyp7a1 and downregulating PCSK9 and SREBP1.

### 3.3. MDP Administration Mitigated Both Local and Systemic Inflammation in ApoE^−/−^ Mice

**Figure 5:**
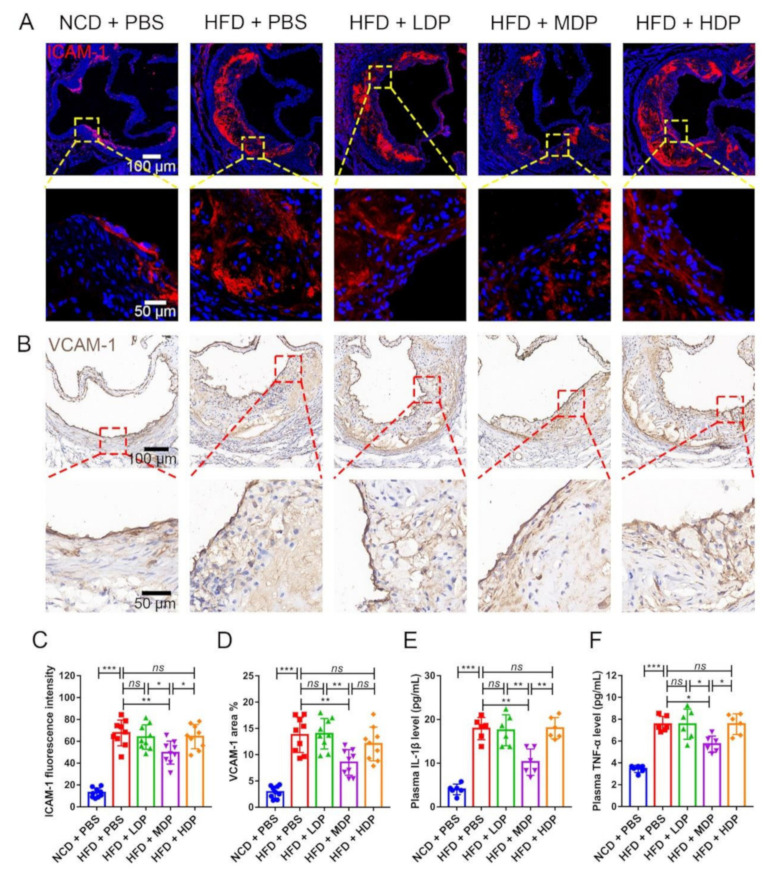
Local and systemic inflammations were reduced in *ApoE^−/−^* mice after oral gavage with middle-dose dual-protein (MDP) nutrition. *ApoE^−/−^* mice were grouped and treated as in Figure 1. (**A**) Representative immunofluorescence staining for ICAM-1 (red) in atherosclerotic lesions. (**B**) Representative immunohistochemical staining for VCAM-1 (brown) in atherosclerotic lesions. (**C**) The fluorescence intensity of ICAM-1 was quantified by Image J software. (**D**) The positive area of VCAM-1 was quantified by Image J software and calculated as the percentage of total lesion area. (**E**,**F**) The circulating levels of interleukin-1β (IL-1β) and tumor necrosis factor-α (TNF-α) were measured by ELISA after gavage with dual-protein nutrition. Data are presented as mean ± SD. *n* = 6 to 9, * *p* < 0.05, ** *p* < 0.01, *** *p* < 0.001, *ns* indicates no significance. Phosphate-buffered saline (PBS), normal-chow-diet (NCD), high-fat-diet (HFD), low-dose dual-protein (LDP), high-dose dual-protein (HDP).

The effect of DP on inflammation was investigated as inflammation has been critically linked to the development of atherosclerosis [3]. ICAM-1, which plays a crucial role in the development and progression of atherosclerosis [1], was inhibited in *ApoE^−/−^* mice treated with MDP (Figure 5A,C); however, no significant difference was detected in the HFD + LDP group and HFD + HDP group. Vascular cell adhesion molecule 1 (VCAM-1), which mediates leukocyte–endothelial cell adhesion, has been proven to be associated with atherosclerosis [25]. A similar effect of MDP on suppression of the high-fat-diet-induced protein expression of VCAM-1 was observed in the aortic roots of *ApoE^−/−^* mice (Figure 5B,D). Similarly, compared with the high-fat-diet mice, MDP-treated mice had significantly fewer macrophages in atherosclerotic lesions, as shown by immunofluorescent staining of the macrophage/myeloid associated antigen CD68 (Figure S1A,B).

Circulating proinflammatory cytokines such as IL-1β and TNF-α are involved in the process of atherosclerosis [26]. Compared with HFD + PBS, the levels of plasma IL-1β and TNF-α in MDP + PBS were decreased by 42.74% and 24.16%, respectively (Figure 5E,F). However, no significant difference was observed in the other two groups treated with DP.

### 3.4. Oral Gavage with MDP Ameliorated Intestinal Barrier Function in ApoE^−/−^ Mice

**Figure 6:**
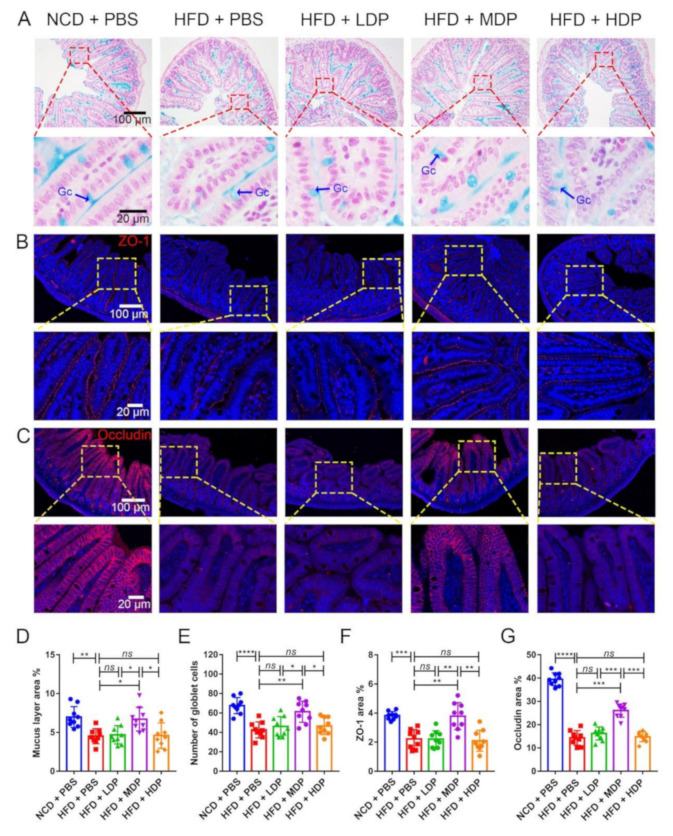
Middle-dose dual-protein (MDP) nutrition regulated intestinal barrier function of *ApoE^−/−^* mice. *ApoE^−/−^* mice were grouped and treated as in Figure 1. (**A**) Representative Alcian blue staining of ileum sections. The mucin layer and the goblet cells (Gc) were visualized under the microscope. (**B**) Representative immunohistochemical staining for ZO-1 (red) in the ileum. (**C**) Representative immunohistochemical staining for occludin (red) in the ileum. (**D**,**E**) Quantitative analysis of the images from A. (**F**,**G**) Quantitative analysis of images from B and C. Data are presented as mean ± SD. *n* = 9, * *p* < 0.05, ** *p* < 0.01, *** *p* < 0.001, **** *p* < 0.0001, *ns* indicates no significance. Phosphate-buffered saline (PBS), normal-chow-diet (NCD), high-fat-diet (HFD), low-dose dual-protein (LDP), high-dose dual-protein (HDP).

A recent study has shown that intestinal barrier dysfunction could cause bacteria and endotoxins to enter the human body, leading to increased susceptibility to inflammatory diseases in the body, which in turn lead to the onset of atherosclerosis [27]. Intestinal barrier function is regulated by the mucus layer and tight junctions of the intestine [28]. Therefore, the effect of DP on the mucin layer area and expression levels of the major tight junction proteins in the intestine was explored. The mucus and goblet cells in the ileum were assessed by the Alcian blue binding assay (Figure 6A). The area of the mucus layer treated with MDP increased significantly compared with that in the HFD + PBS group (Figure 6D). Consistent with the mucus layer area result, the number of mucus-secreting goblet cells appeared to increase (Figure 6E). In addition, the expression levels of the tight junction proteins, including ZO-1 and occludin, were significantly reduced in the HFD + PBS group but upregulated in the MDP + PBS group. (Figure 6B,C,F,G). However, LDP and HDP had no significant effect on the expression of ZO-1 and occludin.

### 3.5. The Effect of DP Treatment on Gut Microbiota

**Figure 7:**
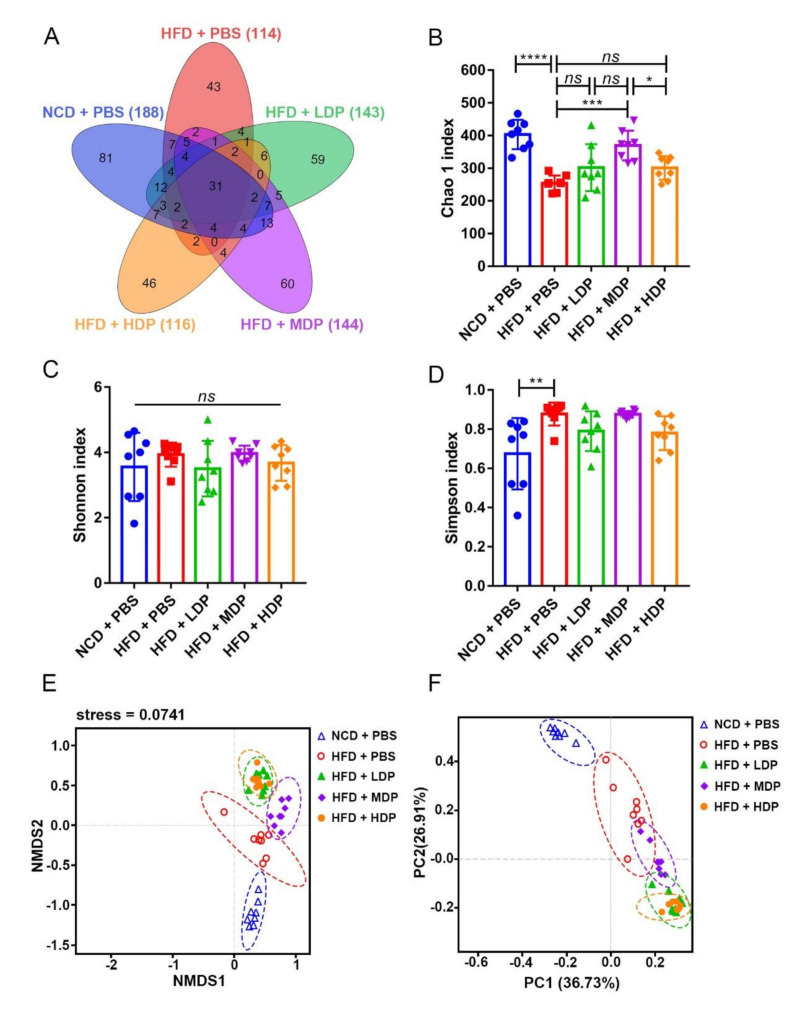
Cecal content diversity analysis after oral gavage with dual-protein (DP) nutrition. *ApoE^−/−^* mice were grouped and treated as in Figure 1. (**A**) Unique and shared intestinal operational taxonomic units (OTUs) of each group are shown in the Venn diagram. (**B**–**D**) Alpha diversity (Chao1 index, Shannon index, Simpson index); beta diversity indicated by nonmetric distance scaling (NMDS) (**E**) of weighted UniFrac distance and principal coordinates analysis (PCoA) plot (**F**). Data are presented as mean ± SD. *n* = 8, * *p* < 0.05, ** *p* < 0.01, *** *p* < 0.001, **** *p* < 0.0001, *ns* indicates no significance. Phosphate-buffered saline (PBS), normal-chow-diet (NCD), high-fat-diet (HFD), low-dose dual-protein (LDP), middle-dose dual-protein (MDP), high-dose dual-protein (HDP).

Related research has shown that changes in the composition and diversity of gut microbiota are closely related to the occurrence of chronic low-grade inflammation and atherosclerosis [29], so we investigated the effect of DP treatment on the gut microbiota composition. Under the similarity threshold of 97%, 423 OTUs were obtained. The average of OTUs for each group and the overlap are shown by a Venn diagram (Figure 7A). The Chao1 index of alpha diversity indicated that treatment with MDP substantially increased the heterogeneity of the gut microbiota compared with the HFD + PBS group (HFD + MDP: 369.5 ± 45.2, HFD + PBS: 254.3 ± 23.66) (*p* < 0.001) (Figure 7B). However, no significant difference emerged in the Shannon index among the groups (Figure 7C). The Simpson index suggested that the gut microbiota diversity was remarkably increased by the high-fat diet (Figure 7D). The weighted UniFrac nonmetric multidimensional scaling analysis revealed that the gut microbiota communities of the HFD + MDP group were markedly different from those of the HFD + PBS group, the HFD + LDP group, and the HFD + HDP group. However, the gut microbiota communities of the HFD + LDP group aggregated more closely to those of the HFD + HDP group (Figure 7E). In line with the NMDS, similar results appeared in PCoA based on the decision UniFrac distance (Figure 7F).

**Figure 8:**
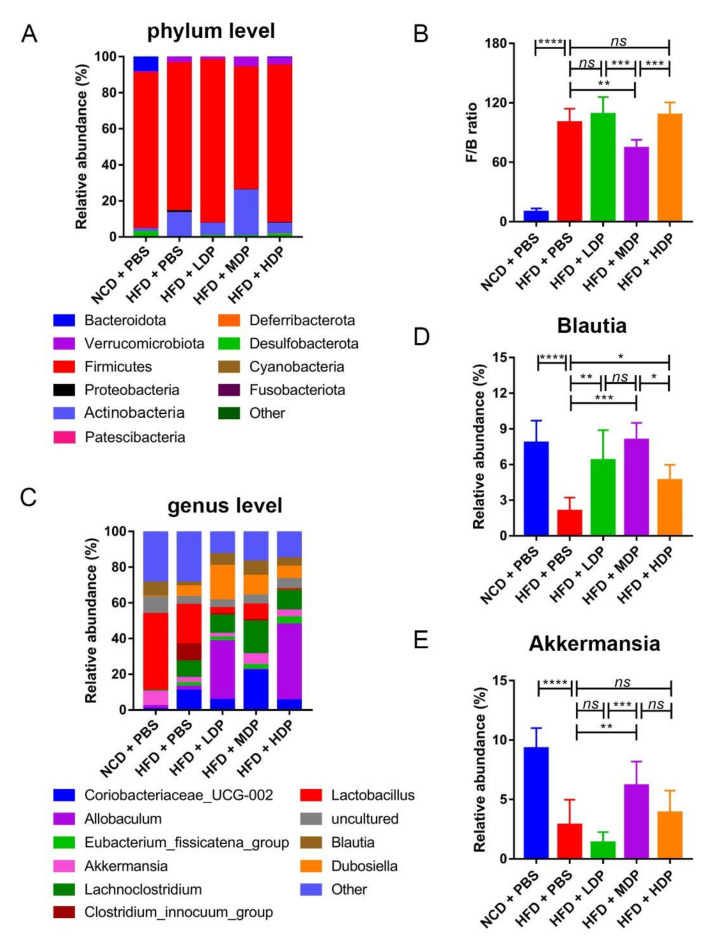
Relative abundance of bacteria at the phylum and family level. *ApoE^−/−^* mice were grouped and treated as in Figure 1. (**A**) Bacterial taxonomic profiling of intestinal bacteria from different groups at the phylum level. (**B**) Ratio of Firmicutes to Bacteroidota (F/B) in the five groups. (**C**) Bacterial taxonomic profiling of intestinal bacteria from different groups at the genus level. (**D**,**E**) Relative abundance of *Blautia* and *Akkermansia* in the five groups. Data are presented as mean ± SD. *n* = 8, * *p* < 0.05, ** *p* < 0.01, *** *p* < 0.001, **** *p* < 0.0001, *ns* indicates no significance. Phosphate-buffered saline (PBS), normal-chow-diet (NCD), high-fat-diet (HFD), low-dose dual-protein (LDP), middle-dose dual-protein (MDP), high-dose dual-protein (HDP).

At the phylum level, compared with the HFD + PBS group, the HFD + MDP group exhibited a marked reduction in the Firmicutes/Bacteroidota ratio by 25.74% (*p* < 0.05) compared to the HFD + PBS group, which is often related to the risk of CVD including obesity [30] (Figure 8A,B). The abundance of Actinobacteria in the HFD + MDP group (25.42 ± 8.47%) (*p* < 0.05) was higher than that in other groups. At the genus level, the relative abundance of *Blautia* in the HFD + MDP group was significantly higher than that in the HFD + PBS group (HFD: 2.08 ± 1.14%, HFD + MDP: 8.07 ± 1.44%) (*p* < 0.01) (Figure 8C,D). Notably, the relative abundance of *Akkermansia*, which is negatively correlated with CVD [31], was higher in the HFD + MDP group than in the other three groups fed with a high-fat diet, as shown in Figure 8E.

## 4. Discussion

Previous studies have proven that soy protein isolate and whey protein can reduce the risk factors of AS, including lipid metabolism and inflammation, and DP consisting of soy protein isolate and whey protein had a better effect than single components [10,32,33,34]. However, the DP intake remains ambiguous. In this study, we found that long-term supplementation of 0.67 g/kg/day DP effectively alleviated atherosclerosis lesions in *ApoE^−/−^* mice induced by high-fat diet by setting different doses of DP. In addition, by measuring the basic physiological parameters of mice, including body weight and food intake, it was confirmed that oral gavage DP as a dietary intervention method has no side effects on the health of *ApoE^−/−^* mice.

Dyslipidemia is a risk factor for AS [3]. Steatosis is associated with risk factors for atherosclerosis such as obesity, diabetes, and dyslipidemia [35]. Therefore, we performed liver Oil Red S staining and observed that MDP significantly reduced high-fat-diet-induced lipid deposition. Our results were similar to those reported by Oliveira and Bortolotti [36,37], who showed that soy protein or whey protein supplements can improve liver steatosis in patients. Interestingly, DP supplements did not change the size of adipocytes. In addition, the role of protein in regulating blood lipid is ambiguous. A study on Zucker diabetic rats revealed that after 13 weeks of supplementation with different forms of whey protein, plasma HDL-C level was significantly increased, but plasma TC level was also significantly increased [38]. However, a study on patients with mild hypertension showed contrary results that 8 weeks of intake of whey protein significantly reduced plasma TG and TC levels [39]. In this study, MDP significantly decreased plasma TG and LDL-C levels but did not reduce the elevated plasma TC and HDL-C levels induced by high-fat diet. Therefore, the effect of MDP on blood lipid levels may depend on the intervention method and the study object. Although the level of LDL-C in the HFD + HDP group decreased, atherosclerosis was not ameliorated in HFD + HDP group. There are many factors that affect lipid metabolism, such as lipid deposition and changes in plasma TG, TC, HDL-C, and LDL-C levels. Only LDL-C was decreased in the HDP group, while lipid deposition was decreased in the MDP group, along with plasma TG and LDL-C levels, indicating that the MDP was better than LDP and HDP in regulating lipid metabolism. Since the liver is a pivotal organ involved in lipid metabolism, we assessed whether MDP improved lipid profiles via regulating relevant signal molecules in the liver. Although LXRα is highly expressed in metabolically active tissues such as liver, kidney, and adipose tissue and regulates cholesterol reversal by regulating downstream genes ABCA1 and ABCG1 [23], the DP did not affect the mRNA expression of LXRα, ABCA1, and ABCG1, which was different from previous reports [12]. MDP supplementation significantly upregulated the mRNA expression of Cyp7a1, a rate-limiting enzyme in the classical bile acid synthesis pathway, in the liver but did not change the mRNA expression of Cyp27a1, a key enzyme in the alternative pathway. Similar results have been reported by Zhang, H. [40]. In addition, MDP supplementation downregulated PCSK9 expression [30], which is an independent predictor of high plasma LDL-C levels. MDP supplementation also downregulated the expression of SREBP1 and SREBP2, which are important genes involved in regulating cholesterol metabolism. Therefore, MDP may promote cholesterol metabolism by upregulating the expression of Cyp7a1 and downregulating PCSK9, SREBP1, and SREBP2. Of course, there may be other potential pathways involved in lipid metabolism.

Inflammation is another risk factor for AS [3]. In the early stages of atherosclerosis, damaged or inflamed endothelial cells begin to express selective adhesion molecules on their surface, specifically ICAM-1, which binds to various types of white blood cells before transmigration into the intima [41]. Inflammatory mediators such as macrophage colony-stimulating factor can increase the expression of macrophage scavenger receptors and promote the uptake of modified lipoproteins to form lipid-containing macrophages, thereby promoting the proliferation and migration of white blood cells and smooth muscle cells in the lesion area [41]. Therefore, anti-inflammatory intervention is widely used in the treatment of AS. For example, Li et al. reported that the anti-atherosclerotic roles of *Akkermansia* depend on its anti-inflammatory effects [28]. This was consistent with our results, which showed that administration of MDP significantly reduced the expression of adhesion molecules ICAM-1 and VCAM-1 induced by high-fat diet, along with lowered aortic infiltration of macrophages, and alleviated atherosclerotic lesions in *ApoE^−/−^* mice. In this study, we found that MDP supplementation significantly reduced plasma IL-1β and TNF-α levels. Similar changes in inflammatory factors have been observed in Crohn’s disease patients, and supplementation with whey and soy protein can alleviate inflammation [33].

Maintaining normal intestinal barrier function is essential to prevent metabolic diseases, and a high-fat diet usually leads to an increase in intestinal permeability [28]. Kan Xiao reported that whey protein concentrate supplementation upregulated intestinal tight junction protein expression in piglets, thus regulating intestinal barrier function [42]. The intestinal chemical barrier is mainly the mucus layer covering the intestinal epithelium, which is rich in various mucins and forms a hydrated gel layer covering the mucosal surface to prevent the adhesion of harmful bacteria [43]. The intestinal physical barrier is mainly composed of epithelial cells and their tight junctions, which are multiprotein complexes composed of occludin, ZOs, etc. A loss of tight junction protein leads to increased intestinal permeability [44]. In our study, MDP supplementation significantly increased mucus layer area and mucus-secreting goblet cells compared with the high-fat-diet group. In addition, immunofluorescence staining analysis of tight junction protein showed that the expression levels of ZO-1 and occludin protein in the ileum were significantly increased compared with the HFD + PBS group, indicating functions of MDP in improving intestinal barrier function damaged by high-fat diet.

In recent years, some studies have shown that the changes of intestinal microbes were closely related to the occurrence of diabetes, obesity, and cardiovascular diseases [29,45], suggesting that the gut microbiota can be used as a potential therapeutic target to reduce the risk of metabolic diseases. So far, there are few reports on whether DP supplementation affects the gut microbiota diversity in *ApoE^−/−^* mice [46]. Our results showed that MDP significantly increased the Chao1 index of alpha diversity, and beta diversity also demonstrated that MDP could induce significant changes in the gut microbiota composition, suggesting that MDP could improve the intestinal dysbacteriosis caused by high-fat diet to some extent. The phylum Firmicutes/Bacteroidota ratio was increased by the high-fat diet, whereas it was significantly decreased by the supplementation of MDP. It has been reported that an increase in the Firmicutes/Bacteroidota ratio could contribute to hypertension and heart failure [47]. However, although the abundance of Firmicutes in the HFD + MDP group showed a decreasing trend (73.68 ± 6.74%) compared to the HFD + PBS group, there was no significant change in the abundance of Firmicutes between the NCD + PBS group and HFD + PBS group (NCD + PBS: 86.43 ± 5.015%, HFD + PBS: 82.03 ± 9.28%), which is consistent with the study of Xie et al. [48]. *Akkermansia* was reported to be capable of protecting against atherosclerosis by preventing inflammation [28]. In this study, high-fat-diet-induced inflammation in *ApoE^−/−^* mice could be inhibited by MDP supplementation; meanwhile, the relative abundance of *Akkermansia* in the HFD + MDP group was higher than that in the HFD + PBS group, suggesting that the *Akkermansia* may be involved in the MDP-mediated anti-inflammatory responses. Notably, we found that MDP significantly increased *Blautia* abundance, and the increase in this beneficial bacteria genus is negatively correlated with obesity [49]. Compared with other groups, the abundance of *Actinobacteria* in the HFD + MDP group was the highest, indicating that MDP may reduce AS by increasing the abundance of *Actinobacteria*. Based on these findings, MDP may prevent atherosclerosis caused by the high-fat diet by altering the diversity and composition of gut microbiota.

## 5. Conclusions

In summary, the present study demonstrated that long-term supplementation of DP at a dose of 0.67 g/kg/day can reduce high-fat-diet-induced atherosclerosis in *ApoE^−/−^* mice, which was accompanied by improvements in vascular plaque, lipid metabolism, inflammatory response, intestinal barrier function, and gut microbiota structure. However, there are significant differences in physiological and metabolic functions between mice and humans [50]. Further human studies are required to confirm our results, which will not only provide new dietary intervention strategies for the prevention of atherosclerosis but also contribute to the development and application of functional foods.

## Figures and Tables

**Table 1:** Primer sequences used for qPCR analyses.

Gene	Primer Sequence (Sense 5′–3′)	Primer Sequence (Antisense 5′–3′)
LXRα	GCGCTTTGCCCACTTTACTG	TCTTCAGCAAGGCGATCTGG
ABCA1	TGGGCTCCTCCCTGTTTTTG	GTCACTTTCATGGTCGCTGC
ABCG1	CTACGGCTTGGACCGAGAAG	ACCTCTCAGCCCGGATTTTG
Cyp7a1	CTGGGGGATTGCTGTGGTAG	GCACAGCCCAGGTATGGAAT
Cyp27a1	AGGCACAGGAGAGTACGGAG	AAGTCCCAAAGGAGGTTGTCC
PCSK9	ATCACCGACTTCAACAGCGT	GCCCTTCCCTTGACAGTTGA
SREBP1	GCCATCGACTACATCCGCTT	GTCTCCACCACTTCGGGTTT
SREBP2	GGCGATGAGCTGACTCTCGG	CCTCCAGGGAAGGAGCTACAA

## Data Availability

The data presented in this study are available on request from the corresponding author. They have not yet been entered into a repository.

## Supplementary Materials

The following supporting information can be downloaded at: https://www.mdpi.com/article/10.3390/nu14040855/s1, Figure S1: Local inflammation was reduced in *ApoE^−/−^* mice after oral gavage with middle-dose dual-protein (MDP). *ApoE^−/−^* mice were grouped and treated as in Figure 1. (A) Representative immunofluorescence staining for macrophage-specific antigen CD68 (red) in atherosclerotic lesions; (B) The fluorescence intensity of CD68 was quantified by Image J software. Data were presented as mean ± SD. *n* = 6 to 9, * *p* < 0.05, ** *p* < 0.01, *** *p* < 0.001, *ns* indicates no significance.
