# Supplementary material for: Dosage of Dual-Protein Nutrition Differentially Impacts the Formation of Atherosclerosis in ApoE−/− Mice
Source: Nutrients. 2022 Feb 18;14(4):855. doi: 10.3390/nu14040855 (PMC8879330; doi:10.3390/nu14040855)
Supplement: Supplementary file 1 [file nutrients-14-00855-s001.zip › nutrients-1588392-supplementary.pdf]

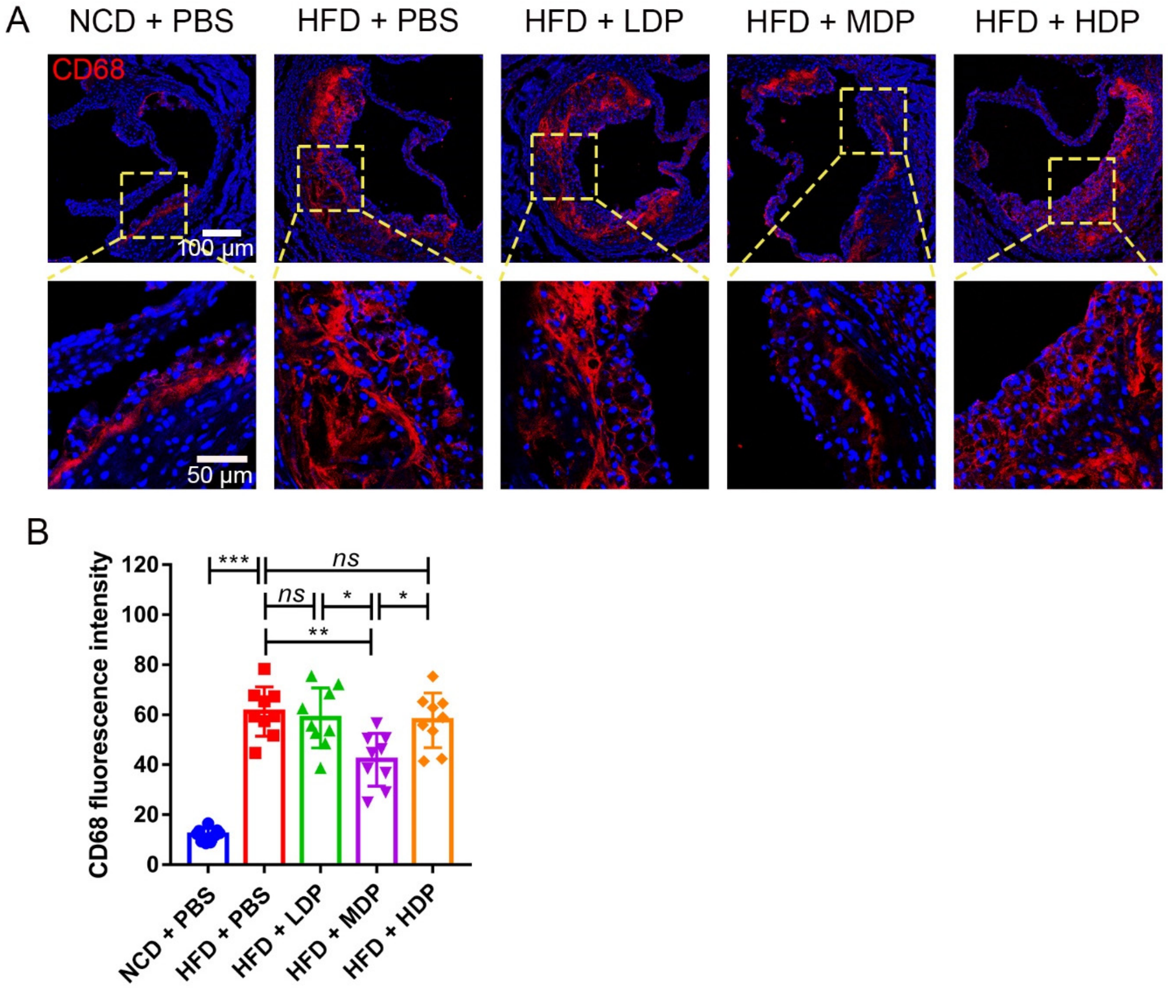

Figure S1. Local inflammation was reduced in *ApoE*$^{-/-}$ mice after oral gavage with middle-dose dual-protein (MDP). *ApoE*$^{-/-}$ mice were grouped and treated as in Figure 1. (A) Representative immunofluorescence staining for macrophage-specific antigen CD68 (red) in atherosclerotic lesions; (B) The fluorescence intensity of CD68 was quantified by Image J software. Data were presented as mean ± SD. n = 6 to 9, *P < 0.05, **P < 0.01, ***P < 0.001, *ns* indicates no significance. Phosphate-buffered saline (PBS), normal-chow-diet (NCD), high-fat-diet (HFD), low-dose dual-protein (LDP), high-dose dual-protein (HDP).
